# Physical Fitness Level in 9–11-Year-Old Italian Children Is Affected by Body Mass Index and Frequency of Sport Practice but Not by Peak Height Velocity and Relative Age Effect

**DOI:** 10.3390/sports14010010

**Published:** 2026-01-03

**Authors:** Mattia Varalda, Alexandru Nicolae Ungureanu, Alberto Coassin, Nicolò Maffei, Damiano Li Volsi, Paolo Riccardo Brustio, Corrado Lupo

**Affiliations:** 1 School of Exercise & Sport Sciences, University of Turin, 10126 Turin, Italy; mattia.varalda@unito.it (M.V.); albycoa1998@gmail.com (A.C.); maffei.nicolo@gmail.com (N.M.); 2 NeuroMuscular Function|Research Group, School of Exercise and Sport Sciences, University of Turin, 10126 Turin, Italy; alexandru.ungureanu@unito.it (A.N.U.); damiano.livolsi@unito.it (D.L.V.); paoloriccardo.brustio@unito.it (P.R.B.); 3 Department of Life Sciences and Systems Biology, University of Turin, 10123 Turin, Italy; 4 Department of Medical Sciences, University of Turin, 10126 Turin, Italy; 5 Department of Clinical and Biological Sciences, University of Turin, 10043 Turin, Italy

**Keywords:** physical evaluation, prepubescents, motor competence, body mass index, sport participation

## Abstract

This study was aimed at analyzing physical fitness in 9–11-year-old children and verifying whether it is affected by body mass index (BMI), peak height velocity (PHV), quartile distribution (QD), and sport practice (SP), also considering any potential effects of sex. One thousand one hundred forty-three Italian primary school children (50.7% males) underwent anthropometric measurements (body mass, height, and BMI) and physical tests for measuring coordination (Plate Tapping, PT), handgrip strength (HandGrip, HG), lower-limb power (standing long jump, SLJ), low-back flexibility (sit-and-reach, SR), and sprint (20 m sprint, 20 m) skills. A series of analyses of covariance (ANCOVAs) were conducted using age as a covariate to examine differences among subgroups for BMI, PHV, QD, and SP in relation to the different physical tests (i.e., PT, HG, SLJ, SL, 20 m). Sex was included in each model as fixed independent variable. Principally, participants with higher SP and BMI reported higher and lower performance (*p* < 0.001) in SLJ, SR, and 20 m tests, respectively. Differently, for higher BMI levels, higher HG performance was reported (*p* < 0.001). PHV and QD had isolated effects, whereas no effect emerged for PT. Sex interactions were found only for SP subcategories in SR (*p* ≤ 0.001, ES range = 0.74–1.30). Although physical performance in 9–11-year-old (non-competitive, pre-puberty) Italian students does not seem to be characterized by involuntary factors (such as PHV and QD), substantial opposite trends seem to exist for voluntary factors (such as BMI and SP), thus suggesting how an adequate lifestyle and physical activity could crucially lead to valuable fitness benefits.

## 1. Introduction

The evaluation of physical fitness (PF) is a key component to better understanding physical capacity in children, which is considered crucial for promoting health [1,2,3] and encouraging active leisure, including participation in recreational activities [4]. International guidelines recommend that children and adolescents aged 5–17 years engage in at least an average of 60 min per day of Moderate-to-Vigorous-intensity Physical Activity (MVPA) [5]. Nevertheless, daily MVPA has tended to decrease among pubertal children over the last several decades, leading to a decline in PF and capacity [6]. For instance, in Italy, only one in three children aged 6–9 years achieves adequate levels of MVPA, whereas one child in four spends 4 h or more watching television daily [7]. According to the literature [8], the primary school years appears to be a particularly critical period for the development of PF and motor capacities, due to the influence of both somatic growth and functional maturation and the practice of several sport activities and disciplines on these two components [3].

However, from a wider perspective, many other factors can influence PF in children. These include chronological factors such as chronological year age and quartile distribution (QD; children born between January and March, April and June, July and September, and October and December are classified into quartile 1, Q1; quartile 2, Q2; quartile 3, Q3; and quartile 4, Q4, respectively) for calculating the relative age effect (RAE) and body mass index (BMI); indices of maturation such as peak height velocity (PHV); and contextual factors (e.g., the school environment and attendance, physical activity at school, and sport practice, SP). In fact, the RAE (i.e., potential age differences within an annually age-grouped cohort, with their consequences) [9] phenomenon highlights an evident bias in many youth sport-specific contexts [9,10] in both boys and girls, thus determining alteration in talent identification [11,12,13]. Moreover, factors such as BMI and PHV are investigated in relation to physical capability development for different range ages (i.e., prepubertal, pubertal, adolescent samples), sex (i.e., females, males), socioeconomic factors, and deficits in executive function [14]. School-related physical activity (PA) and sport practice (SP) can improve well-being and increase positive mental health in children and adolescents [7,15].

However, studies conducted by Drenowatz et al. [16] examined differences in various components of PF in Austrian primary school children, aged 6–11 years old, analyzing possible correlations with BMI and the RAE. In relation to BMI, overweight/obese children displayed lower PF and capacities, except for upper body strength. Further, the improvement in fitness tests alongside increasing age was less pronounced in overweight/obese children compared to their normal-weight peers [17]. Relatively older primary school children displayed better performance in strength and power, speed, agility, and object control, while differences in cardiorespiratory endurance were less pronounced, highlighting the need to consider individual differences in the evaluation of children’s fitness [18]. However, even regardless of potential RAEs, PF differences have been commonly attributed to heterogenous maturations and cognitive skills, as well as the behavioural, motor, social, and emotional development [18] occurring at prepubertal and pubertal ages [19,20]. In fact, RAE consequences are mostly evident during childhood: children born in the first six months of their year of birth are also more likely to continue their participation in sports and additional activities useful for further improving their physical skills [21].

Regarding SP, several studies have investigated the relationship between this factor and PF through questionnaire administration for assessing PA levels (i.e., Physical Activity Questionnaire for older Children, PAQ-C), but not through fitness tests [7]. In particular, regarding Italian children’s PA levels, Lupo et al. [7] evaluated the impact of PA level on PF by controlling for individual characteristics. Their study was able to demonstrate the association between PA level and scores in some fitness tests (i.e., upper-limb strength, balance, low-back flexibility, cardiorespiratory fitness, lower-limb muscle power, and sprint ability). In addition, children’s BMI was positively associated with upper-limb strength and lower-limb muscle power, whereas the opposite trend emerged for cardiorespiratory fitness, sprint ability, and balance. Finally, no absolute relationship emerged between PA and the fitness tests considered, since this depends on the test type and children’s characteristics [7].

However, the relationship between PF and several individual factors is still unclear, and, despite the fact that independent variables such as BMI, PHV, and SP have already been investigated using physical tests administered to the same sample, no information has been provided for RAE calculated on a non-competitive 9–11-year-old sample.

Thus, this study aimed to evaluate 9–11-year-old Italian children performing PF (assessed through fitness skills including Plate Tapping (PT) for hand–eye coordination, handgrip (HG) for upper-limb strength, standing long jump (SLJ) for lower-limb muscle power, sit-and-reach (SR) for low-back flexibility, and 20 m sprint (20 m) for sprint ability), verifying whether performance in each test is influenced by different BMI, PHV, QD, and SP subcategories, also considering potential differences between sexes. As a consequence, it was hypothesized that test performance would be influenced by BMI, PHV, QD, and SP, even when considering differences between sexes.

## 2. Materials and Methods

### 2.1. Participants

One thousand one hundred forty-three children (50.7% males, age 10.4 ± 0.6 yrs, weight 39.4 ± 10.0 kg, height 142.3 ± 9.1 cm, BMI 19.2 ± 3.7 kg/m^2^) aged 9–11 years old from 5 primary schools (i.e., 18 school sections; 65 classes in total) in Vercelli city and its surroundings (Piedmont, Northwest Italy) participated in the present cross-sectional study. Regarding the sampling procedure, all schools recruited for the study were part of the same institutional and regional district and received the same delivery of educational curricula to include a homogenous socio-cultural environment. Before data collection, the institutional review board of the University of Turin approved the study (protocol #0353400; 15 April 2025). A class session to provide preliminary information about the aim of the study was provided to participants. As inclusion criteria, the analyses of the study have been applied only for healthy participants with no locomotor impediments had to be present on the days of the test battery to take part in the experimental measurements. Consequently, participants absent on the days of data collection were excluded. The entire sample of participants was analyzed according to the following subgroups: sex (male, female); age category (i.e., 9, 10, or 11 yrs); BMI (low, adequate, or high BMI with respect to the specific national range for age and sex) [17]; PHV (>6 months before, 0–6 months before, 0–6 months after, >6 months after the average sample time for the PHV) [22]; birth dates, which were used to calculate the RAE (1st quartile: January–March; 2nd quartile: April–June; 3rd quartile: July–September; 4th quartile: October–December); and SP (0, 1–2, >2 weekly practice).

### 2.2. Methods

**Figure 1:**
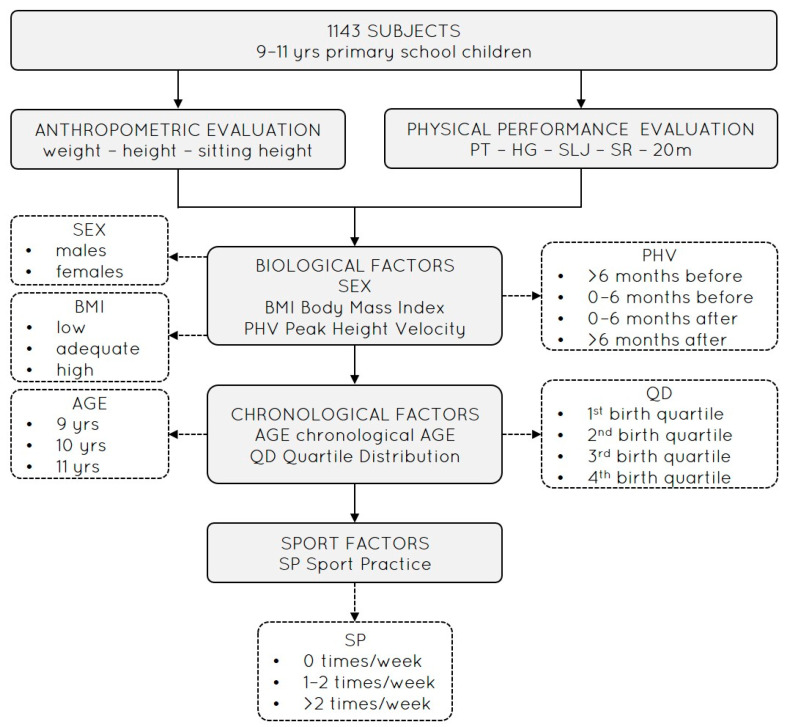
Experimental study design with subjects, evaluations, factors, and subgroups. Notes: PT, Plate Tapping; HG: handgrip; SLJ: standing long Jump; SR: sit-and-reach; 20 m: 20 m sprint.

Each class was tested during 3 consecutive weekly Physical Education (PE) lessons, always at the same time. Lesson times were different for each class, according to weekly school hour planning (i.e., 1st hour from 8.30 to 9.30, 2nd hour from 9.30 to 10.30, 3rd hour from 10.30 to 11.30, and 4th hour from 11.30 to 12.30). The first test session included the registration of participants’ birthday (to calculate the participants’ chronological age and quartile classification within the single birth year to calculate the RAE), SP (i.e., number of individual weekly training sport sessions, recorded by individually interviewing each participant), and anthropometric parameter evaluation (i.e., body mass, stature, sitting height). The other two test sessions included the evaluation of PF. In particular, hand–eye coordination, upper-limb strength, and lower-limb muscle power (i.e., PT, HG, SLJ) were assessed in the second session, while low-back flexibility and sprint ability (i.e., SR, 20 m) in the third session. All physical tests are recognized as part of the Eurofit battery (mean test–retest ICC: PT = 0.87, HG = 0.94, SLJ = 0.93, SR = 0.96, 20 m = 0.94) [23] and were performed twice with 30 s (i.e., PT, HG, SR) and 60 s (i.e., SLJ, 20 m) rest in between. Individual performances at each test station were interspersed by 8–10 min; then, the best score of each test was considered for statistical analysis. Test sessions were carried out in the school gyms with similar surfaces and spaces in order to guarantee equal test circumstances and satisfactory safety measures. All measurements were performed by the same two expert investigators (>2000 participants previously tested using the same tests) following a standardized test protocol. During the test session, participants were encouraged to perform as best as they could in each trial. The experimental study design diagram is reported in Figure 1.

### 2.3. Procedures

#### 2.3.1. Anthropometric Parameters

The anthropometric evaluation was carried out under the same conditions (i.e., lesson day, time, wearing t-shirt and shorts, barefoot). Stature, body mass, and sitting height were measured to assess anthropometric parameters. Stature and sitting height were evaluated using a portable stadiometer (MZ10042, ADE, Hamburg, Germany) with an accuracy of 0.01 cm. Body mass was measured using an electronic scale (876, Seca, Hamburg, Germany) with an accuracy of 0.1 kg. BMI was calculated according to the following formula: body mass (kg)/stature squared (m^2^). PHV was calculated with the Mirwald equations (specified for sex, with results expressed in years) to obtain the maturity offset value [22].

#### 2.3.2. Hand–Eye Coordination

The Plate Tapping test was performed to assess hand–eye coordination. A table with adjustable height, yellow discs (diameter of 20 cm), and a blue rectangle (side × side, 20 × 30 cm), placed equidistant between both discs, were used to perform PT. The table height was adjusted so the subject stood comfortably in front of the rectangle and the discs. The non-preferred hand was placed on the rectangle, and the child moved the preferred hand as fast as possible back and forth between the discs, with the non-preferred hand put in the middle. The action was repeated for 25 full cycles (i.e., 50 taps) and the time of the trial (s) was recorded once the participant touched the yellow disc for the last tap; a timer (HS-30, Casio, Tokyo, Japan) was used to record the time, to the nearest 0.01 s.

#### 2.3.3. Upper-Limb Strength

Handgrip strength was measured using a dynamometer (EH101, Gripx, Frankfurt am Main, Germany), calibrated previously to tests by means of a check against a known weight. According to Lupo et al. [7], participants stood with their dominant arms constantly vertical and close to the body during the test. The palm was not flexed on the wrist joint. The subjects were required to exert maximal strength on the dynamometer (i.e., maximum voluntary contraction) using the dominant hand. The dynamometer scale indicated handgrip strength in kg, with an accuracy of 0.1 kg.

#### 2.3.4. Lower-Limb Muscle Power

The standing long jump test was performed to assess lower-limb power. According to Lupo et al. [7], children jumped as far as possible off the stand, trying to land with both feet and maintaining equilibrium once they landed [7]. The test measured the distance jumped (cm) (i.e., the distance between the last heel mark and the take-off line) using a measurement to the nearest 0.5 cm.

#### 2.3.5. Low-Back Flexibility

Hip and low-back flexibility was ascertained with a sit-and-reach box (Standard flexibility, Baseline, New York, NY, USA) to perform the sit-and-reach test. According to Lupo et al. [7], the child was instructed to place one hand on the other and slowly reach forward as far as possible while keeping the knees extended. The hands were kept aligned evenly as the subject reached forward along the box’s surface. The final position of the fingertips on the ruler was recorded (expressed in cm) to the nearest 0.5 cm.

#### 2.3.6. Sprint Ability

Sprint ability was measured with a dual infrared reflex photoelectric cell system (Chrono Time, Globus, Codognè, Treviso, Italy) to record the time taken to perform a 20 m linear sprint test, to the nearest 0.01 s. The instrument had previously been calibrated to tests by means of a specific reset procedure. Subjects began the test from a standing start with their preferred foot forward, 0.5 m from the first timing gate and the front toe on the start line. Once ready, they sprinted as fast as possible until crossing the stop line. Thus, subjects decelerated after the photoelectric cell system, according to Lupo et al. [24]. The time recorded when the subject passed the start and stop line was considered as the performance time (s).

### 2.4. Statistical Analysis

A series of analyses of covariance (ANCOVAs) were conducted using age as a covariate to examine differences among subgroups for BMI, PHV, QD, and SP in relation to the different physical tests (i.e., PT, HG, SLJ, SL, 20 m). Sex was included in each model as a fixed independent variable. The inclusion of age as a covariate allowed for controlling for potential confounding effects related to developmental differences among participants. Partial Eta Square (η_p_) values were calculated to describe the main effect size. When necessary, Bonferroni-adjusted post hoc pairwise comparisons were performed. Cohen’s d effect sizes (ES) with 95% were used to describe the practical meaningfulness of the differences in mean values. Absolute ES values were evaluated according to the following thresholds: <0.2 = trivial, 0.2–0.6 = small, 0.7–1.2 = moderate, 1.3–2.0 = large, and >2.0 = very large. All the statistical analyses were carried out using the statistical package R (version 4.5.1-13 June 2025) with the packages lme4 (version 1.1-37), emmeans (version 2.0.0), and effectsize (version 1.0.1).

## 3. Results

**Figure 2:**
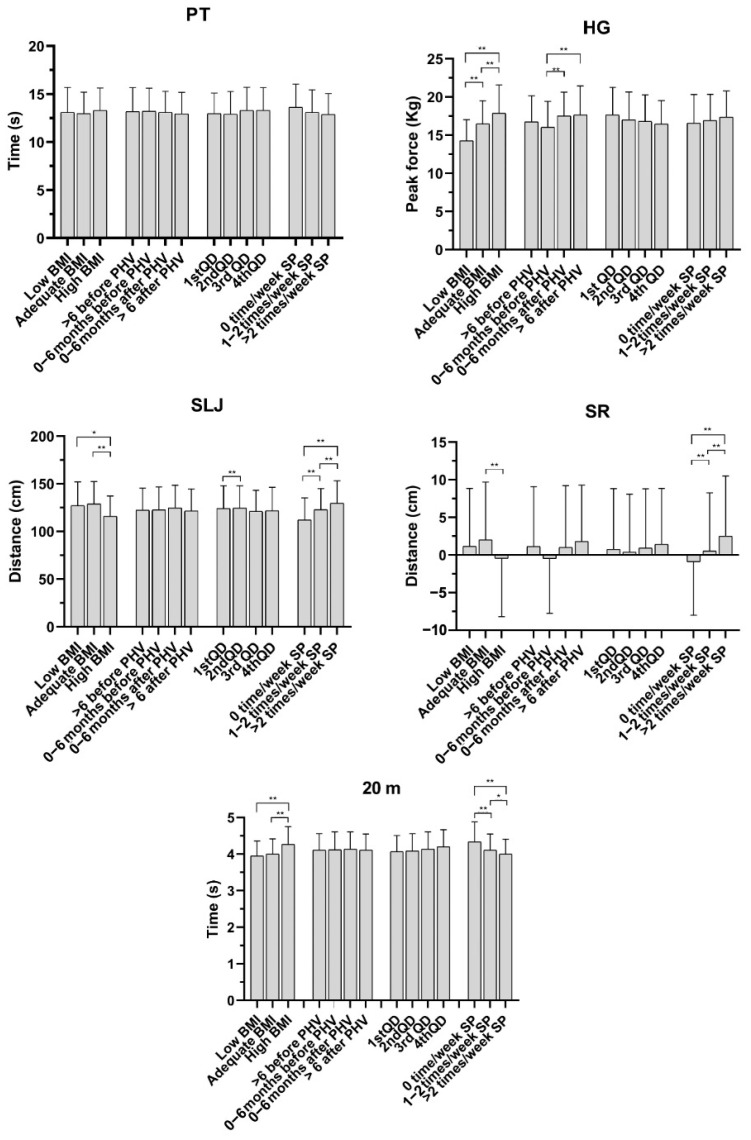
Pairwise comparisons of BMI (low, adequate, high), PHV (>6 months before, 0–6 months before, 0–6 months after, >6 months after), QD (1st, 2nd, 3rd, 4th quartile), and SP (0, 1–2, >2 times/week) in relation to each physical test (PT, HG, SLJ, SR, 20 m). (Notes: * *p* ≤ 0.01, ES range 0.2–0.3; ** *p* ≤ 0.001, ES range ≥ 0.3.)

Main effects of the independent variable (BMI, PHV, QD, SP) and consequent subcategory post hoc effects (Figure 2) were reported for each physical test, including effects for each physical test except for PT.

For HG, main effects emerged for BMI (F = 22.91, *p* ≤ 0.001, ηp = 0.041) and PHV (F = 5.84, *p* ≤ 0.001, ηp = 0.016). Considering BMI, all post hoc comparisons were significant (low vs. adequate, *p* < 0.001, ES = 0.8; β = −2.40; low vs. high, *p* < 0.001, ES = 1.2, β= −3.92; ade-quate vs. high, *p* < 0.001, ES = 0.5, β = −1.52). On the other hand, post hoc analyses of PHV reported differences between 0–6 months before and 0–6 months after (*p* = 0.001, ES = 0.3, β = −1.13), and between 0–6 months before and > 6 months after (*p* ≤ 0.001, ES = 0.3, β = −1.22).

Regarding SLJ, main effects emerged for BMI (F = 18.42, *p* ≤ 0.001, ηp = 0.033), RAE (F = 3.01, *p* = 0.03, ηp = 0.008), and SP (F = 13.92, *p* < 0.001, ηp = 0.025). In BMI, post hoc effects emerged between low and high BMI (*p* = 0.003, ES = 0.5, β = 10.05) and between adequate and high BMI (*p* ≤ 0.001, ES = 0.6, β = 13.02). In SP, effects emerged for 0 times/week vs. 1–2 times/week (*p* ≤ 0.001, ES = 0.5, β = −10.54), 0 times/week vs. >2 times/week (*p* ≤ 0.001, ES = 0.7, β = −16.53), and 1–2 times/week vs. >2 times/week (*p* ≤ 0.001, ES = 0.3, β = −5.98), whereas for QD, only differences between Q1 and Q2 were reported (*p* = 0.007, ES = 0.3, β = −6.80).

Considering SR, main effects emerged for BMI (F = 8.13, *p* ≤ 0.001, ηp = 0.014) and SP (F = 21.73, *p* ≤ 0.001, ηp = 0.038). Post hoc analysis reported only an effect for BMI, with a difference between adequate and high BMI (*p* ≤ 0.001, ES = 0.3, β =2.05). Differently, for SP, all post hoc comparisons were significant (0 times/week vs. 1–2 times/week, *p* ≤ 0.001, ES = 0.6, β = −1.77; 0 times/week vs. >2 times/week, *p* < 0.001, ES = 0.6, β = −3.77; 1–2 times/week vs. >2 times/week, *p* ≤ 0.001, ES = 0.3, β = −1.99).

Similarly to SR, for the 20 m sprint test, main effects emerged for BMI (F = 18.12, *p* ≤ 0.001, ηp = 0.03) and SP (F = 10.42, *p* ≤ 0.001, ηp = 0.018). However, BMI post hoc analyses reported effects between low and high (*p* ≤ 0.001, ES = 0.7, β = −0.28), as well as adequate and high (*p* ≤ 0.001, ES = 0.6, β = −0.25), BMI values. For SP, all post hoc comparisons were significant (0 times/week vs. 1–2 times/week, *p* ≤ 0.001, ES = 0.5, β = 0.22; 0 times/week vs. >2 times/week, *p* ≤ 0.001, ES = 0.7, β = 0.31; 1–2 times/week vs. >2 times/week, *p* = 0.006, ES = 0.2, β = 0.09).

Finally, no effect was reported for sex interactions with respect to each independent variable, except for SP in the SR test (F = 4.63, *p* = 0.009, ηp = 0.008), where female subjects regularly showed higher SR values than their male counterparts for each SP level (Table 1).

## 4. Discussion

The present study aimed at verifying whether performance on physical tests (PT, HG, SLJ, SR, 20 m) is affected by BMI, PHV, QD, and SP factors. It also hypothesized the presence of potential effects between sexes (controlling for participants’ age). Although the main aim of the study was mostly fulfilled, especially for effects of BMI and SP on almost all proposed tests, only the SR test was affected by sex differences (significantly higher values for female participants) for each SP subcategory. As a consequence, the experimental hypothesis can be considered to be partially confirmed. In fact, PF was firstly affected by different BMI and SP levels, whereas the impact of different PHV and QD subcategories was almost absent. In addition, only SP levels, and only for one physical test (i.e., SR), showed sex-related differences, highlighting a countertendency with respect to previous studies, in which 20 m and HG were different between sexes [7]

This study is a useful representation of the PF status of children living in Northwest Italy. The robust statistical approach, in which age was applied as a controlling variable, underlined how “voluntary” factors (i.e., those modifiable by the subjects) have a strong impact on fitness, whereas PHV and GS, which can be considered as “involuntary” (i.e., innate), were not determinant. In addition, the exclusive consideration of sex in the presence of previously emerging significance for an independent variable deeply highlights how SR should be considered a sex-dependent physical test, in which female participants fundamentally seem to display greater mobility in comparison with their male counterparts [25].

According to the test outcomes, BMI was the most significant factor (in four tests out of five, with a small-to-moderate ES); it was significant in the upper-limb strength, lower-limb muscle power, low-back flexibility, and sprint ability tests (Figure 2). In particular, the trends between BMI and HG differed with respect to those for SLJ and the 20 m sprint test. In fact, although higher BMI determines lower individual fitness level, as confirmed by non-satisfactory performance in lower-limb muscle power and sprint tests, the opposite trend emerged for upper-limb strength, with significant results of pairwise comparisons and a moderate ES. However, these heterogenous findings for BMI and HG are in line with a previous study [7] evaluating fitness levels in Italian primary school children, where negative relationships emerged between BMI and lower-limb muscle power and sprint, and positive ones were seen with respect to upper-limb strength [7]. Therefore, this highlights how an inadequate BMI (above the upper threshold of the optimal range) can limit adequate performance in physical tests but can favour higher strength. Moreover, we can speculate whether the HG result would be less important if the sample was characterized by a wider, more negative fitness scenario. Finally, children with adequate BMI levels reported the best SR outcomes compared to the results of high-BMI subcategories. We can assume that this effect could be expected. Nevertheless, this finding is in contrast with previous works in which low-back flexibility was not affected by different BMI levels [7,17].

The fitness outcome in relation to the different levels of SP was also in line with the expected hypotheses. In fact, SP effects emerged for three of five tests. Differently from BMI, the upper-limb strength test was not related to SP, while the lower-limb muscle power, low-back flexibility, and 20 m sprint were represented by positive and linear trends, in which higher physical outcomes emerged for higher SP levels (with small-to-moderate ESs), thus confirming the results of a previous and similar study [26] on children of a similar age, in which better SLJ and 20 m performance was achieved by the highest SP category, although this was not the case for SR. Conversely, in our study, SP factors for the SR test represented a unique case, as we saw differences not only with regard to sex, but even for sex interactions, thus confirming the results of another study [27] in which it was confirmed that females are regularly better than males in terms of SR performance. In particular, these effects are also characterized by a moderate ES, which strengthens the solidity of this finding.

GD showed results not completely consistent with the literature, as evident RAEs were not reported. In fact, the presence of significance only for the Q1–Q2 comparison in SLJ seems to be due to a divergent distribution of the data (also confirmed by a small ES), instead of being a clear trend. Therefore, considering that the RAE is almost absent, this sample of subjects seems to be in contrast with previous studies, in which significant relationships emerged between children born in different quartiles of the school year and fitness for similar age ranges [28,29] and fitness tests [30]. In particular, according to Gadžić et al., significant RAEs exist in physical variables in both sexes in five out of eight motor tests (i.e., Eurofit test battery), with a large ES [30]. Likewise, Jarvis et al. [29] investigated Welsh primary school children of a similar age and found significant RAE differences for some Fundamental Movement Skill (FMS) tests (i.e., catch, overhand throw, kick) in boys only [29]. As a consequence, further research should be encouraged to better understand RAEs in in samples of non-athlete subjects.

Furtherly, PHV subcategories reported weak effects in relation to children’s results in fitness tests. In fact, only in two cases for HG results did advanced PHV subcategories show better results with respect to the 0–6 months before subcategory, albeit with a small ES. Differently, in a previous study conducted on children of a similar mean age (although with a larger age range) [31], HG differed according to PHV levels, even when controlling for other factors. However, the same trend was not found for the other physical tests, although several fitness tests were found to be influenced by PHV [32]. Moreover, previous findings in the literature refer to sport-specific samples, which could differ from non-athlete individuals [32]. As consequence, the real effect of PHV on PF results remains clear; we can speculate that, similarly to GS, the peculiarities of each sample (e.g., athletes/non-athletes) can result in different trends.

Finally, the absence of PT effects seems to demonstrate that this physical test is not influenced by different levels in the BMI, SP, or PHV and GS categories, thus resulting in a contrast with previous studies in which the outcomes of this physical test performed in primary school students were different between the sexes [27].

In terms of experimental limitations, potential biases could emerge as the anthropometric and PF evaluations were performed at different times of the day (i.e., according to the PE lessons planned for each class). Also, the frequency of physical activity was assessed by a questionnaire which does not reflect information such as the intensity, duration, or type of physical activity and could be affected by some biases (e.g., in self-reporting sport practice). Also, the use of the Mirwald equation (error ± 1 year) for maturity offset in a narrow age range may not be so reliable and may only represent an estimation instead of a direct measurement [22]. Additionally, the selected physical tests, although feasible in terms of space and time and referring to the highly consolidated Eurofit battery, were not representative of all physical capacities (e.g., the SR test can assess low-back flexibility but not overall flexibility, and the 20 m linear sprint measures sprint ability but not agility), and the lack of an assessment of cardiorespiratory fitness could be a significant gap for a more complete PF evaluation. Finally, data were collected in a restricted area in Northwest Italy and without any consideration of ethnicity or academic performance, limiting any generalization to the entire nation or continent. For these reasons, future longitudinal research could be conducted on a wider series of fitness tests while also evaluating other youth categories (i.e., pubertal children), with participants coming from other parts of Italy or Europe, and simultaneously involving other growth factors in analyzing PF outcomes.

## 5. Conclusions

The present study was able to highlight how PF in 9–11-year-old Italian children, assessed through coordination (PT), handgrip strength (HG), lower-limb power (SLJ), low-back flexibility (SR), and sprint (20 m) skills, is substantially influenced by different BMI levels and grades of SP. In particular, regardless of sex, all physical tests, excepting PT, were performed differently by children with different BMI levels (with a moderate ES). Similarly, lower-limb power (SLJ), low-back flexibility (SR), and sprint (20 m) skills differed in relation to three different SP levels. Conversely, only sporadic effects emerged for the PHV and GS subcategories, thus providing a new contribution to the literature, but without clarifying contrasting findings reported for these factors in non-athlete subjects.

Therefore, the take-home message of the present study is that PF in 9–11-year-old (non-competitive, pre-puberty) Italian students differs according to BMI and SP level, thus highlighting that an adequate lifestyle and level of fitness (i.e., adequate BMI, frequent sport practice) should be regularly promoted [33]. Conversely, weaker tendencies were verified for PHV and GS, suggesting that these factors do not substantially characterize non-athlete pre-puberty subjects [28,29]. In other words, we can speculate that, while physical performance in 9–11-year-old Italian students does not seem to depend on “involuntary” factors (such as PHV and QD), it is substantially modifiable by “voluntary” ones (such as maintaining a healthy BMI and high involvement in SP), strengthening the importance of the above-mentioned lifestyle-related message in determining valuable benefits of physical fitness, starting in childhood [26].

## Figures and Tables

**Table 1:** Sex interactions (estimated means ± standard errors, coefficient intervals; β scores, *p* values, Cohen’s effect sizes) in the SR test in relation to SP subcategories.

SP Subcategories	Females (cm)	Males (cm)	β	*p*	ES
0 time/week	1.37 ± 0.67 (−0.25 to 2.31)	−3.64 ± 0.73 (−5.07 to −2.20)	5.02	<0.001	0.74
1–2 times/week	4.14 ± 0.39 (3.29 to 4.87)	−2.88 ± 0.39 (−3.66 to 2.12)	7.02	<0.001	1.03
>2 times/week	7.03 ± 0.55 (6.12 to 8.29)	−1.77 ± 0.53 (−2.65 to −0.57)	8.80	<0.001	1.30

Descriptive data (means ± standard deviations) of the subjects’ characteristics for age subgroups (9, 10, 11 years old), independent variables (BMI, PHV, QD, SP), and each physical test is reported in the Supplementary Materials (Table S1).

## Data Availability

The raw data supporting the conclusions of this article will be made available by the authors on request.

## Supplementary Materials

The following supporting information can be downloaded at: https://www.mdpi.com/article/10.3390/sports14010010/s1, Table S1: Descriptive data (means ± SD) of the subjects’ characteristics, reported for age subgroups (9, 10, 11 years old), independent variables (BMI, PHV, QD, SP), and each physical test.
