# Supplementary material for: Physical Fitness Level in 9–11-Year-Old Italian Children Is Affected by Body Mass Index and Frequency of Sport Practice but Not by Peak Height Velocity and Relative Age Effect
Source: Sports (Basel). 2026 Jan 3;14(1):10. doi: 10.3390/sports14010010 (PMC12845823; doi:10.3390/sports14010010)
Supplement: Supplementary file 1 [file sports-14-00010-s001.zip › sports-3947330-supplementary.pdf]

Table S1. Descriptive data (means±SD) of the subjects' characteristics, reported for age subgroups (9, 10, 11 years old), independent variables (BMI, PHV, QD, SP), and each physical test.

Group	Subgroup	N subjects	Age (yrs)	Body mass (kg)	Stature (cm)	Sitting height (cm)	BMI (kg/m^2)	PHV (yrs)	QD (%)				SP (wp)	PT (s)	HG (kg)	SLJ (cm)	SR (cm)	20 m (s)
									1st q	2nd q	3rd q	4th q						
Females	9 yrs (n= 143)	145	9.6±0.2	35.0±7.3	137.0±6.2	114.4±4.7	18.6±3.0	11.6±0.5	22.9	29.8	21.8	25.5	1.7±1.2	13.2±3.0	15.0±2.7	110.7±21.7	2.7±7.1	4.4±0.5
	10 yrs (n=302)	293	10.5±0.3	39.8±9.9	142.8±10.5	120.0±10.8	19.2±3.6	11.8±0.6	22.2	27.8	28.4	21.6	1.9±1.3	13.0±2.5	16.9±3.4	119.9±23.7	4.4±7.5	4.2±0.6
	11 yrs (n=119)	125	11.1±0.2	43.4±9.8	148.3±7.3	125.1±9.8	19.6±3.6	11.8±0.5	23.4	24.5	25.0	27.2	2.0±1.5	12.8±3.2	18.7±3.9	123.3±25.4	6.1±7.1	4.0±0.4
	All females (n=564)	563	10.4±0.6	39.4±9.7	142.5±9.7	119.7±10.1	19.1±3.5	11.7±0.5	22.8	27.4	25.1	24.7	1.9±1.3	13.0±2.8	16.8±3.6	118.3±24.0	4.3±7.4	4.2±0.5
Males	9 yrs (n=155)	159	9.6±0.2	35.0±7.5	137.0±6.1	114.1±9.2	18.5±3.2	13.1±1.0	27.3	26.8	27.3	18.7	2.0±1.2	13.8±2.8	15.9±3.1	122.1±20.9	-2.6±6.0	4.2±0.5
	10 yrs (n=292)	287	10.5±0.3	39.7±10.5	142.8±6.9	119.6±8.0	19.3±4.0	13.4±0.5	26.9	25.4	25.9	21.8	2.1±1.2	13.1±2.4	17.3±3.5	126.9±24.6	-2.8±6.5	4.0±0.5
	11 yrs (n=132)	134	11.2±0.2	43.1±10.9	146.0±11.3	122.2±9.0	21.1±14.9	13.5±0.6	25.4	24.9	25.4	24.3	2.0±1.3	12.9±2.6	18.6±4.0	134.1±25.0	-2.1±7.5	4.0±0.4
	All males (n=579)	580	10.4±0.6	39.2±10.3	141.9±8.6	118.7±9.1	19.5±7.9	13.3±0.7	26.6	25.7	26.2	21.4	2.0±1.3	13.2±2.6	17.2±3.6	127.2±24.1	-2.6±6.6	4.0±0.5
All subjects (n=1143)		1143	10.4±0.6	39.3±10.0	142.2±9.2	119.2±9.6	19.3±6.1	12.5±1.0	24.7	26.5	25.7	23.1	1.9±1.3	13.1±2.7	17.0±3.6	122.8±24.4	0.9±7.8	4.1±0.5

Notes: BMI: Body Mass Index; PHV: Peak Height Velocity; QD: quartile distribution; SP: Sport Practice; PT, Plate Tapping; HG: HandGrip; SLJ: Standing Long Jump; SR: Sit and Reach; 20 m: 20 m sprint.
